# Structural and Thermomagnetic Properties of Gallium Nanoferrites and Their Influence on Cells In Vitro

**DOI:** 10.3390/ijms241814184

**Published:** 2023-09-16

**Authors:** Marta Orzechowska, Katarzyna Rećko, Urszula Klekotka, Magdalena Czerniecka, Adam Tylicki, Dariusz Satuła, Dmytro V. Soloviov, Anatoly I. Beskrovnyy, Arkadiusz Miaskowski, Beata Kalska-Szostko

**Affiliations:** 1 Doctoral School of Exact and Natural Sciences, University of Bialystok, K. Ciołkowskiego 1K, 15-245 Białystok, Poland; 2 Faculty of Physics, University of Bialystok, K. Ciołkowskiego 1L, 15-245 Bialystok, Poland; k.recko@uwb.edu.pl (K.R.); d.satula@uwb.edu.pl (D.S.); 3 Faculty of Chemistry, University of Bialystok, K. Ciołkowskiego 1K, 15-245 Białystok, Poland; u.klekotka@uwb.edu.pl (U.K.); kalska@uwb.edu.pl (B.K.-S.); 4 Faculty of Biology, University of Bialystok, K. Ciołkowskiego 1J, 15-245 Białystok, Poland; m.siemieniuk@uwb.edu.pl (M.C.); atyl@uwb.edu.pl (A.T.); 5 European Molecular Biology Laboratory, Notkestraße 85, 22607 Hamburg, Germany; dimkaupml@gmail.com; 6 Frank Laboratory of Neutron Physics, Joint Institute for Nuclear Research, Joliot-Curie 6, 141980 Dubna, Russia; beskr@nf.jinr.ru; 7 Department of Applied Mathematics and Computer Sciences, University of Life Sciences in Lublin, Akademicka 13, 20-950 Lublin, Poland; arek.miaskowski@up.lublin.pl

**Keywords:** superparamagnetic nanoparticles, X-ray and neutron diffraction, small angle neutron scattering, calorimetric measurements, Mössbauer spectroscopy, in vitro cell culture

## Abstract

Magnetite and gallium substituted cuboferrites with a composition of Ga_x_Fe_3−x_O_4_ (0 ≤ x ≤ 1.4) were fabricated by thermal decomposition from acetylacetonate salts. The effect of Ga^3+^ cation substitution on the structural and thermomagnetic behavior of 4–12 nm sized core-shell particles was explored by X-ray and neutron diffraction, small angle neutron scattering, transmission electron microscopy, Mössbauer spectroscopy, and calorimetric measurements. Superparamagnetic (SPM) behavior and thermal capacity against increasing gallium concentration in nanoferrites were revealed. The highest heat capacity typical for Fe_3_O_4_@Ga_0.6_Fe_2.4_O_4_ and Ga_0.6_Fe_2.4_O_4_@Fe_3_O_4_ is accompanied by a slight stimulation of fibroblast culture growth and inhibition of HeLa cell growth. The observed effect is concentration dependent in the range of 0.01–0.1 mg/mL and particles of Ga_0.6_Fe_2.4_O_4_@Fe_3_O_4_ design have a greater effect on cells. Observed magnetic heat properties, as well as interactions with tumor and healthy cells, provide a basis for further biomedical research to use the proposed nanoparticle systems in cancer thermotherapy (magnetic hyperthermia).

## 1. Introduction

Accompanying the rapid progress in nanotechnology toward biofunctionalized particles, tremendous efforts have been made in recent years to optimize core and core/shell magnetic nanoparticles (MNPs). The core/shell type nanoparticles can be broadly defined as comprising a core (inner material) and a shell (outer layer material). Because both terminologies, “shell@core” and “core/shell”, became equally popular in the literature, the authors of the paper decided to use the former. Currently, magnetic nanoparticles are of particular interest as a diagnostic and therapeutic tool, which, due to their small size, can overcome most biological barriers, allowing their diffusion and distribution into most tissues [1,2]. Well-controlled preparation of pure and doped nanomagnetites allows for modeling their properties, which results in many benefits in various fields of science [3,4,5]. Notably, different types of shell@core nanoparticles, surface stabilizer@inorganic nanoparticles, and, more specifically, magnetic core materials coated with other inorganic materials, are extensively studied because of their wide biomedical applications [6,7]. In addition, biocompatible polymers, such as polyethylene glycol, are used extensively with a view to increasing the biocompatibility of the core [8]. Superparamagnetic iron oxide nanoparticles (SPIO NPs) can transform both cancer diagnostics [9,10,11] and therapeutics [12,13]. The interest in them stems from relatively easy synthesis, good corrosion resistance, high magnetization, and relatively low cytotoxicity [14]. The US Federal Drug Administration (FDA) has approved iron oxide nanoparticles for use in human therapeutics and diagnostics [15,16].

Surface modification and functionalization is an essential aspect of SPIO NP synthesis. Thermal decomposition is a well-known method for the successive synthesis of ferrite core–shell nanoparticles [17]. Such a “heating-up” method involves the decomposition of precursors in the presence of organic surfactants to produce the desired particles. The most commonly used non-magnetic precursors are acetylacetonates and surfactants. The impacts of the surfactant quantity and quality on the growth regime of iron oxide nanoparticles are extremely important. Usually, disperse SPIO NPs with sizes from 3 to 20 nm are prepared at 265 °C in the presence of Fe(acac)_3_, phenyl ether, 1,2-hexadecanediol as a reducing agent, oleic acid, and oleylamine [18]. This process offers yield quantity, particle size control, fine size distribution, crystallinity, and dispersibility to synthesized shell@core nanoparticles. A simplified notation of the composition of nanoparticles was adopted in this work, i.e., M@Ga_x_ and Ga_x_@M, where M means magnetite with the formula Fe_3_O_4_, while x means a dopant of Ga in gallium ferrite.

Magnetites doped with bi- and trivalent cations form a group of innovative materials [19,20,21,22,23,24,25]. Among these, gallium nanoferrites seem to be promising candidates for use in magnetic hyperthermia [12].

Because of their simple crystal structure and high tendency to form homogeneous compounds, gallium-modified magnetites represent an ideal system to study the influence of ionic order on the thermomagnetic features of nanoparticles [26]. A large family of ferrite binary oxides with the spinel structure (Fd-3m no. 227) is characterized as a ferrimagnetic with strong dependence of magnetic properties on the state of chemical order and on the cation distribution between the two crystallographically distinct tetrahedral (Td) and octahedral (Oh) sites. Notably, the crystal ordering for the system of interest is defined according to Formula (1).

(1) $$\left[{\left(Ga_{x-\varepsilon }^{3+}\right)}_{Td}{\left(Ga_{\varepsilon }^{3+}\right)}_{Oh}\right]\left[{\left(\;Fe_{1-\left(x-\varepsilon \right)}^{3+}\right)}_{Td}{\left(Fe^{2+},\;Fe_{1-\varepsilon }^{3+}\right)}_{Oh}\right]O_{4},$$

**Figure 1:**
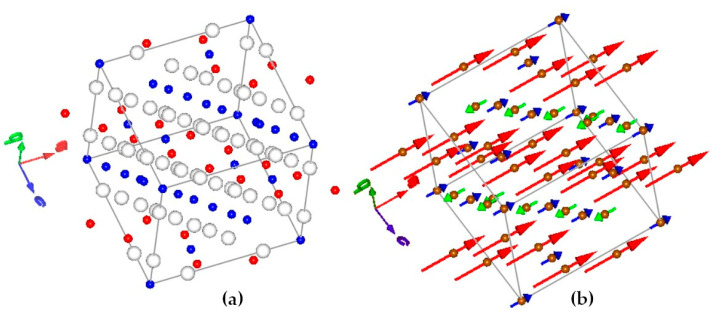
Structural (**a**) and magnetic (**b**) ordering in M@Ga_0.6_ (10 K). In the left panel, the blue dots represent the Td sublattice while the red dots correspond to the Oh sublattice. In the right panel, the red arrows illustrate Fe^2+^ magnetic moments, while the blue and green arrows are related to Fe^3+^ moments.

It can be easily seen that any ε condition leads to the inverse spinel structure of Ga_x_Fe_3−x_O_4_ (for simplicity, hereinafter referred to as Ga_x_). Simultaneously, under the ε=x condition, Ga will only occupy the Oh site, while the ε=0 condition leads to total Td site preference (see, respectively, red and blue dots shown in Figure 1a). One of the problems of contemporary nanomaterial physics currently being solved is the creation of magnetic nanoparticles that conclusively exhibit magnetism that is weak enough to prevent agglomeration but strong enough to preserve superparamagnetic fluctuations up to temperatures well above 300 K. The magnetic ordering of the doped system is illustrated in Figure 1b. The appropriate spin configuration of the gallium nanoferrite is sensitive to the content and location of the Ga dopant [27,28].

The site that is partially occupied by non-magnetic gallium ions will exhibit weaker magnetic exchange interactions. If the gallium prefers any of the spinel sublattice, this will weaken the compensation of the antiferromagnetically oriented magnetic moments of the site Td with respect to Oh (see, respectively, smaller and larger arrows shown in Figure 1b). As a result, the magnetic moment of the single domain (nanoparticle) would increase. The gallium doping of Fe_3_O_4_ may be a simple route of tuning magnetic properties, with direct consequences on superparamagnetic behavior. Gallium-doped magnetite nanoparticles are soft magnetic materials with a phase transition temperature above 350 K [26]. Therefore, it was decided to use the properties of gallium as an admixture of nanoferrites in the presented studies.

Certain properties of magnetic nanocrystals, such as the blocking temperature *T_B_*, magnetic saturation, and permanent magnetization, are all dependent on particle size, but the coercivity of the nanocrystals totally depends on the particle shape because of surface anisotropy effects [29]. By simple modeling both radius and spherical shape dependences versus $T_{B}$ (Equation (2)) it becomes simpler to predict some other parameters that optimize superparamagnetism.

(2) $$T_{B}=\frac{KV}{25k_{B}}=\frac{K\left(\frac{4\pi r^{3}}{3}\right)}{25k_{B}},$$

where: *r*—radius of particle, *V*—volume of spherical particle, $k_{B}$—Boltzmann constant, $T_{B}$—blocking temperature, and *K*—anisotropy constant.

Subsequent modification on the core or shell of spherical nanoparticle influences, in different ways, the magnetic and thermal behavior of the systems.

The superparamagnetic behavior is exhibited by correspondingly small particles. If they are too small, almost all the atoms are on the surface, leading to thermal and magnetic properties strongly modified with respect to the bulk materials. The typical values for spherical particles are about 8–18 nm for Fe_3_O_4_ [27,28,30,31,32]. Notably, with decreasing particle size, the anisotropy energy decreases. For a grain size equal to or lower than a characteristic value, this energy may become even lower than the thermal energy. This implies that the energy barrier for magnetization reversal may be overcome, and the total magnetic moment of the particle can then thermally fluctuate. Finally, the entire spin system may be rotated, and the spins within the single-domain particles remaining magnetically coupled can disclose superparamagnetic behavior. Obviously, the blocking temperature for a magnetic particle increases with increasing size and for a given size increases with decreasing measuring time, and the observation of a superparamagnetic or blocked state then depends on the experimental technique. For example, the techniques used to study the superparamagnetic relaxation are (1) dc susceptibility, where $\tau_{m}$ is estimated to be around $10^{2}\;s$; (2) ^57^Fe Mössbauer spectroscopy with the time window range of $10^{-9}–10^{-7}\;s$; and (3) neutron diffraction with time window of $10^{-12}–10^{-8}\;s$, depending on the type of experiments.

Blocking temperatures determined from RT measurements using the Mössbauer effect (ME) or from neutron diffraction (ND) are significantly higher compared to those from RT dc magnetization (MAG). The reason for this is the much shorter measuring time $\tau_{m}$ in these experimental methods. Assuming the longest possible times of $\tau_{m}^{MAG}=10^{2}\;s$, $\tau_{m}^{ME}=10^{-7}\;s$, and $\tau_{m}^{ND}=10^{-8}\;s$, the superparamagnetic blocking temperatures relate to each other as $\frac{KV}{25.3k_{B}}:\;\frac{KV}{4.6k_{B}}:\;\frac{KV}{2.3k_{B}}$, i.e., 1:5.5:11.

As an example, for Fe_3_O_4_ (for simplicity, hereinafter referred to as M) the characteristic grain diameter below which superparamagnetic relaxations and above which magnetically ordered states are observed is 17 nm for dc susceptibility measurements, while it is 9 nm for Mössbauer spectroscopy experiments, which have a much shorter measuring time [30].

Additionally, in the measurements of saturation magnetization or blocking temperature, it is difficult, in fact, to determine the mass of magnetic nanoparticles in a colloidal ferrifluid suspension (nanoparticles often surrounded by an organic surfactant in a dispersing medium). From a clinical point of view, the toxicity of nanoparticles, their stability under physiological conditions, and their ability to be removed from the body are essential issues [1]. The main disadvantage of nanoparticles in biomedicine is their instability and tendency to form aggregates in aqueous media due to magnetic dipole–dipole and van der Waals interactions [33].

The objective of this work is to study the influence of the location and quantity of gallium dopants on the crystal and magnetic properties of spherical and nearly spherical shell@core nanoparticles of Ga_x_@M and M@Ga_x_ series based on diffraction, spectroscopy, microscopy, and cytotoxic data. In addition, ferrimagnetism, associated with superparamagnetic behavior, is investigated with Mössbauer spectroscopy and low-temperature neutron data. The research is aimed at checking whether the superparamagnetism and specific absorption rate coefficients with high biocompatibility and nontoxicity are in an easily accessible range for potential biomedical applications.

## 2. Results and Discussion

### 2.1. Diffraction Data Analysis

**Figure 2:**
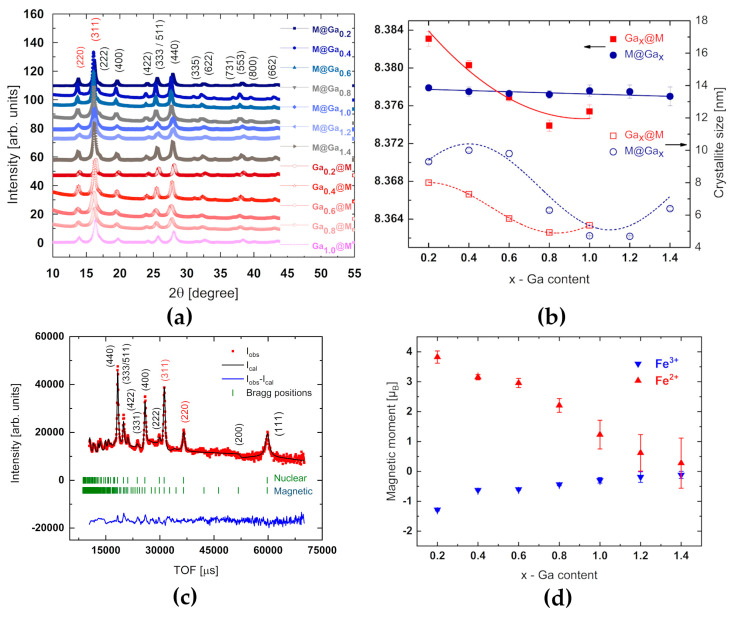
X-ray diagrams collected at room temperature: (**a**) set of both series of nanoparticle patterns with the inset legend of nominal compositions; (**b**) lattice constant and crystalline size versus gallium content, where the curves are guides to the eye only; (**c**) neutron data refinement of M@Ga_0.2_ core-shell system at 10 K and (**d**) iron in-site magnetic moment dependence versus gallium content of M@Ga_x_ series collected at 10 K. The red triangles correspond to Fe^2+^ magnetic moments on the Oh sublattice, while the blue triangles correspond to Fe^3+^ moments occupying the Th sublattice.

Here, the direct evidence is reported of stable crystal structure parameters, independently of gallium contribution and localization at shell@core systems. The patterns of M@Ga_x_ and Ga_x_@M samples with Ga content x = 0.2–1.4 are nanocrystalline. All the samples exhibit a spinel crystal structure. The single-phase samples show all the characteristic reflections of ferrite material with the most intense (311) single reflection and the well visible (333/511) doublet (Figure 2a), which confirm the formation of the cubic spinel structure. The XRD patterns were indexed using the ICSD card No. 98-002-8282.

The fluctuation of the lattice parameter as a function of the increasing Ga content in the Ga_x_@M systems is much stronger than that observed in the M@Ga_x_ series (Figure 2b). Nevertheless, the values of unit cell parameters may indicate the presence of oxygen vacancies, i.e., the maghemite structure. In this case, the weakest vacancy effect for nanosystems with gallium content Ga_x_ = 0.6 is observed. The second, but less likely, explanation concerns the difference in the radii of iron ions and doped cations. The similarity of the Ga^3+^ (62 pm) and Fe^3+^ (64.5 pm) radius, during systematic doping of gallium in the Td sublattice, would lead to negligible contraction of the unit cell. On the other hand, systematic Ga doping in the Oh sublattice, also occupied by Fe^2+^ (78 pm), should result in distinct crystal lattice contraction.

However, the difference in the sensitivity of the unit dimensions of crystal lattice has been confirmed experimentally. On the other hand, the analysis of crystallite size vs. gallium content in both series showed a consistent trend, with the minimum at the range above 0.8 of the gallium content, although the slightly different nature of the relationship in the function of the location of gallium ferrite was observed.

The squares of the modules of geometric structure factors correspond in a simple way to the preference for the occupation of the Td sublattice by gallium (Equation (3)) and to oxygen vacancies (Equation (4)):

(3) $${\left|F_{\left(220\right)}\right|}^{2}={\left[2f_{O}\left(1+cos8\pi X_{O}\right)+f_{Td}\right]}^{2}$$

(4) $${\left|F_{\left(311\right)}\right|}^{2}={\left[-f_{O}\left(cos2\pi X_{O}+2cos6\pi X_{O}+cos10\pi X_{O}+sin2\pi X_{O}-2sin6\pi X_{O}+sin10\pi X_{O}\right)+f_{Td}+\sqrt{2}f_{Oh}\right]}^{2}$$

where $X_{O}$ is the $O^{2-}$ position parameter according to Wyckoff notation 32 e: $\left(X_{O},X_{O},X_{O}\right)$. According to X-ray data, the $X_{O}$ parameter oscillates around 0.25 (see Table 1).

Due to the opposite dependence of the atomic scattering factors, Fe:Ga in X-ray diffraction (starting from the straight scattering case: 26:31) to the relationship of the bond coherent neutron-scattering length Fe:Ga = 9.45:7.288 fm [34], Formula (3) is sensitive primarily to the increasing gallium contribution in the Td sublattice. Such a model would be associated with an increasing X-ray signal and a decreasing signal from neutron scattering. Formula (4) is rather weakly sensitive to cation distribution. On the other hand, due to the oxygen bond coherent neutron-scattering length equal to 5.805 fm, there is the problem of comparable contributions of the oxygen (~4 × 5.805) and cationic terms in Equation (4); thus, oxygen vacancies will manifest by constant or increasing (311) intensity. From the point of view of the X-ray experiment, the scattering contribution from oxygens is almost two times smaller than that from cations. In conclusion, with an increased number of oxygen vacancies, a marked increase in (311) X-ray reflection and a slight decrease or constancy of the (311) neutron peak should be observed.

**Figure 3:**
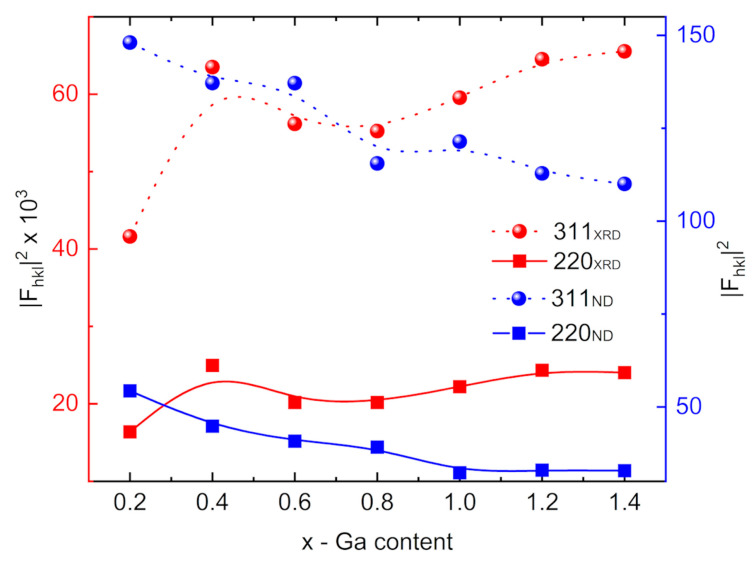
The square structure factors ${\left|F_{\left(hkl\right)}\right|}^{2}$ of structural reflections, (311)—dotted curves, and (220)—solid curves as a function of gallium content for series M@Ga_x_ were obtained from X-ray (red symbols) and neutron (blue symbols) data analysis. The curves are guides to the eye only.

The experimental squares of the behaviour of the modules of geometric structure factors against gallium concentration are shown in Figure 3. During analysis of diffraction data collected at RT and 10 K, X-ray and neutron data were processed simultaneously. Only contributions from nuclear neutron scattering were taken into account for the analysis of the gallium filling trend. Apart from the fluctuation in the range of 0.5–0.8 of the gallium content in nanoferrites, a constant trend is observed, which undoubtedly indicates a preference for the location of gallium in the Td sublattice. The analogous behavior of the reflex (311) indicates the presence of oxygen vacancies throughout the volume of the sample. Refinements of nuclear and magnetic ordering of M@Ga_x_ series collected at 10 K (Figure 2c,d) confirmed most of the properties expected for nanosized ferrites on the base of Fe_3_O_4_. Although the internal structure of gallium nanosystems is based on both a core and a shell to a large extent on magnetite, the neutron magnetic moments associated with the cationic sublattices reveal fundamentally different values (Figure 2d). The magnetic signal from the Th sublattice is nearly 50% weaker than the signal from the Oh sublattice. This missing magnetic signal, similar to a frozen state of disordered spins, may indicate the formation of a spin-glass phase. Any such effects intensify when coupled with the effect of thermal vibrations, which are inevitable at RT. To avoid this, the magnetic ordering was analyzed at the lowest possible temperature of 10 K. Notably, such effects have already been observed in the case of fine particles of ferrimagnetic oxides such as γ-Fe_2_O_3_ [35,36,37,38]. As the particle size decreases, the surface-area-to-volume ratio increases, as does the percentage of atoms (spins) located on the surface. The magnetic properties of the surface of a particle may differ significantly from the magnetic properties of its core, primarily due to: (1) breaking the translational symmetry of the crystal at the particle boundary, and thus, different surroundings of atoms (lower coordination of spins on the surface); (2) existence of broken bonds or gaps between atoms (e.g., oxygen vacancies); and (3) heterogeneity of chemical composition. Although one can expect an ordering of ionic moments within the core of the particle similar to that occurring in the bulk material, the surface of the particles may be characterized by a non-collinear spin structure and spin-glass magnetic behavior [35,39,40,41,42]. The neutron data do not exclude a weak noncollinearity of the spin structure. Moreover, systems with gallium content above x = 1 within single measurement uncertainties are completely magnetically disordered.

### 2.2. TEM Investigations

**Figure 4:**
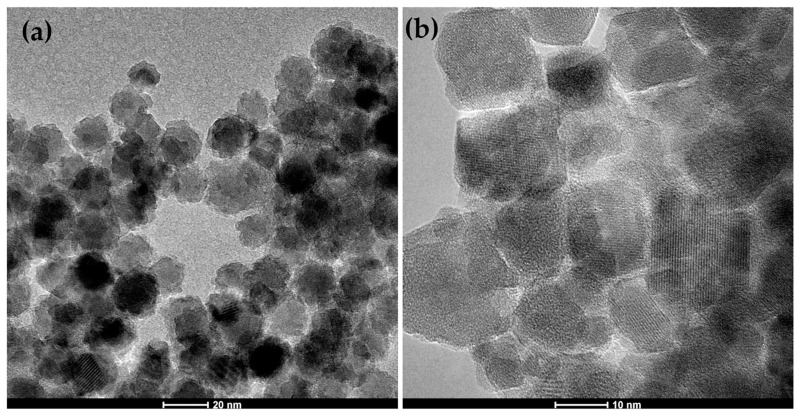
TEM images of nanosystems: Ga_0.2_@M (**a**) and M@Ga_1.0_ (**b**).

The presented TEM images (Figure 4a,b) refer to gallium-doped systems in the shell. Both images show that the nanoparticles tend to agglomerate. The shape is not always spherically symmetrical. Figure 4a shows compact spherical aggregates with irregular surfaces, but they are quite uniform in size. For the whole series with gallium in the core, all images look very similar and the size oscillates around the value of 17 nm [28]. Figure 4b shows nanoparticles of heterogeneous shapes and sizes. For the series with gallium in the shell, the size oscillates within 22 nm. Based on the analysis of the images of the presented systems, it can be concluded that with the increase in the dopant of gallium, the tendency to form spherical clusters decreases, the shape of aggregates is irregular, and the shape heterogeneity and size of individual particles increase.

### 2.3. SANS Data Analysis

Small angle neutron scattering is one of the experimental approaches used in nanoscale structure determination. This is concomitant with the improvement in the description of the inner, core-shell structure of the nanosystems in question. In fact, gallium scatters neutrons weaker than iron. This allows the neutron-scattering length density (*sld*) of the Ga_x_Fe_3−x_O_4_ to be distinguished with respect to magnetite (*sld* = 6.9806) according to Equation (5) in the range from 6.9220 (*x* = 0.2) to 6.5124 (*x* = 1.4).

(5) $$sld_{Ga_{x}Fe_{3-x}O_{4}}=\frac{8\left(xb_{Ga}+\left(3-x\right)b_{Fe}+4b_{O}\right)}{V}\cdot 10^{-6}\left[{Å}^{-2}\right],$$

where *V* is the volume of unit cell [Å^3^]. The scattering function of the isolated particles can be modeled according to the geometry of the *sphere core–shell* model:

(6a) $$I\left(q\right)=\frac{N}{V}{\left[\left(\frac{sld_{c}}{V_{c}}-\frac{sld_{solv}}{V_{solv}}\right)V_{c}\;F(qR_{c})+\left(\frac{sld_{s}}{V_{s}}-\frac{sld_{solv}}{V_{solv}}\right)(V_{s}F(qR_{s})-V_{c}F\left(qR_{c}\right)\right]}^{2}S\left(q\right),$$

where $\frac{N}{V}$ is the density of particles, *sld_i_* is scattering length density, *V_i_* is volume, and *R_i_* is radius of core (*i* = *c*), shell (*i* = *s*), and solvent (*i* = *solv*). Formula (6a) for the *sphere* model simplifies itself to Equation (6b):

(6b) $$I\left(q\right)=\frac{N}{V}{\left[\left(\frac{sld_{c}}{V_{c}}-\frac{sld_{solv}}{V_{solv}}\right)V_{c}\;F(qR_{c})\right]}^{2}S\left(q\right)$$

**Figure 5:**
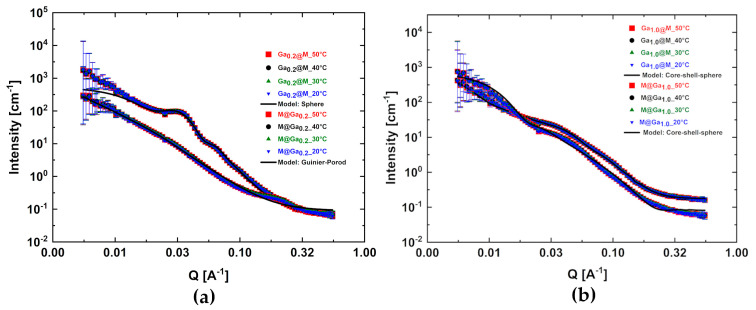
SANS data collected at the temperature range of 20–50 °C for selected core–shell gallium ferrite nanoparticles: (**a**) M@Ga0.2, and Ga0.2@M; (**b**) M@Ga1.0, and Ga1.0@M. The panels illustrate the refinements (solid lines) of the experimental data (symbols).

The problem with an incorrectly selected surface stabilizer was revealed by SANS measurements (see Figure 5a,b). Porod showed that the total small angle scattering from a sample is an invariant irrespective of the way the sample density is distributed. In the case of different matter distribution, it is necessary to use model-independent analysis or a shape-independent model. In theory, the analysis of the so-called *Porod region* allows the calculation of the volume fraction of each component in a complex system given the contrast, or the contrast given the volume fractions. However, in practice, it is difficult to measure the scattering in a wide enough Q range because Porod is a law for scattering at high values of *q* ($q\gg \frac{1}{D}$, where *D* is the size of the scattering aggregates or other scattering objects), if there are sharp boundaries between the aggregates of the system. The law states that at large *q*, the scattering function can be modeled according to Formulas (7) and (8):

(7) $$I\left(q\right)\propto q^{-4},$$

and thus

(8) $$\frac{4\pi^{2}}{q}\underset{q\to \infty }{\lim}\left(I\left(q\right)\cdot q^{4}\right)=\frac{S}{V},$$

where $\frac{S}{V}$ is the *specific surface* of the sample. If we consider two systems, one containing well-dispersed nanoparticles and the second containing partially agglomerated nanoparticles, then the *specific surface* in the first case will be greater than that in the second one, although the density of the neutron-scattering length (*sld*) will remain invariant.

Where the Porod approximation considers the high-*q* limit of scattering, the low-*q* limit can be described using the *Guinier approximation* formulated as follows:

(9a) $$I\left(q\right)=I_{0}\exp\left(-\frac{q^{2}R_{g}{}^{2}}{3}\right),$$

and after transformation into a logarithmic form:

(9b) $$lnI\left(q\right)=\ln\left(I_{0}\right)-\frac{R_{g}{}^{2}}{3}q^{2},$$

and thus the *radius of gyration* of the scattering object, *R_g_*, can be extracted from the slope of a plot of lnI(q) vs. $q^{2}$, bearing in mind that the validity of the approximation is limited to values of $qR_{g}\ll 1$. For example, the radius of gyration of a *sphere or spherical aggregate* is given by $R_{g}{}^{2}=\frac{3}{5}R^{2}$, while for other bodies or agglomerate shapes, this equation should be properly modified. Another aspect is the dimensionality and symmetry of the scattering object. In general, Equation (7) can be written as $I\left(q\right)=Gq^{-m}$ with *G*—Guinier formula and *m*—power related to the object’s symmetry *α*. Consequently, Equation (9a) can be described in the general form of $I\left(q\right)=\frac{G}{q^{\alpha }}\exp\left(-\frac{q^{2}R_{g}{}^{2}}{3-\alpha }\right)$. Notably, a dimensionality parameter (3 − *α*) is thus defined, and for 3D objects such as spheres, *α* = 0, and one recovers the standard Guinier formula *G*. For 2D symmetry, for example, for rods *α* = 1, and for 1D symmetry such as for plates, *α* = 2. A dimensionality parameter (3 − *α*) is thus defined, and is equal to 3 for spherical objects, 2 for rods, and 1 for plates.

Thus, in the case of really significant agglomeration effects, the nanoparticles were treated as being shape independent according to *Guinier–Porod* relations, where:

(10) $$q_{1}=R_{g}^{-1}\sqrt{\frac{\left(m-\alpha \right)\left(3-\alpha \right)}{2}}=R_{g}^{-1}\surd 6,$$

Finally, the size of the scattering objects can be expressed as:

(11) $$D=\frac{G}{R_{g}^{m-\alpha }}exp\left[-\frac{m-\alpha }{2}\right]{\left(\frac{\left(m-\alpha \right)\left(3-\alpha \right)}{2}\right)}^{\frac{m-\alpha }{2}}=\frac{G}{R_{g}^{4}}exp\left[-2\right]{\left(6\right)}^{2}=\frac{4.87G}{R_{g}^{4}}.$$

Figure 5a shows that systems with lower gallium content showed strong dispersity and rapidly agglomerated in dilute diphenyl ether solutions; thus, the *Guinier–Porod* shape-independent model was required for description of *I*(*q*) characteristics. Figure 5b illustrates nanosystems with higher gallium concentrations that could be correctly described by the core–shell model [30]. Sphere radius is treated conventionally as the simple sum of the core radius and shell thickness (Table 2).

Dispersion effects in the case of both analyzed series were visible in the initial part in the area of small wave vectors. Nevertheless, these are more peculiar, interesting characteristics than those obtained in the case of chloride samples synthesized via the Massart method [43,44]. As a rule, dispersity coefficients prefer description with the use of Gaussian or Shultz functions for distribution of radius and thickness, respectively [37]. Due to partial agglomeration, the values of shells were slightly overestimated.

The qualitatively weaker elaborations of the SANS data result (Figure 5) from the need to use simpler shape models, limiting the possibilities of dispersion description. The gallium-doped magnetite nanoparticles keep spherically symmetrical shapes in almost the whole range of the studied admixtures. Therefore, on the basis of Arrhenius’s theory and simple modification of Equation (2), the appropriate superparamagnetic blocking temperature from the SANS data could be estimated (columns 5 and 6 of Table 2). Two different approaches were analyzed for *T_B_* estimation from SANS data. In the first, the dominant nanomagnetite nature of both cores and shells was assumed. Considering the neutron mean measurements and relaxation times, the anisotropy constant $K=1.1\times 10^{4}\frac{J}{m^{3}}$ [45], and the sphere radius, the appropriate *T_B_*_1_ values listed in column 5 were determined. The second approach uses the individual anisotropy constants of nearly isostructural gallium ferrites [26]. Here, the *T_B_*_2_ the values are systematically higher (see column 6 of Table 2), which indicates the determining influence of the shell and the coating agent. The small particle size and low anisotropy constant result in low *T_B_* values. From this point of view, gallium ferrites with a Ga content in the range of 0.6–0.8 are optimal.

### 2.4. Mössbauer Spectroscpy Data Analysis

**Figure 6:**
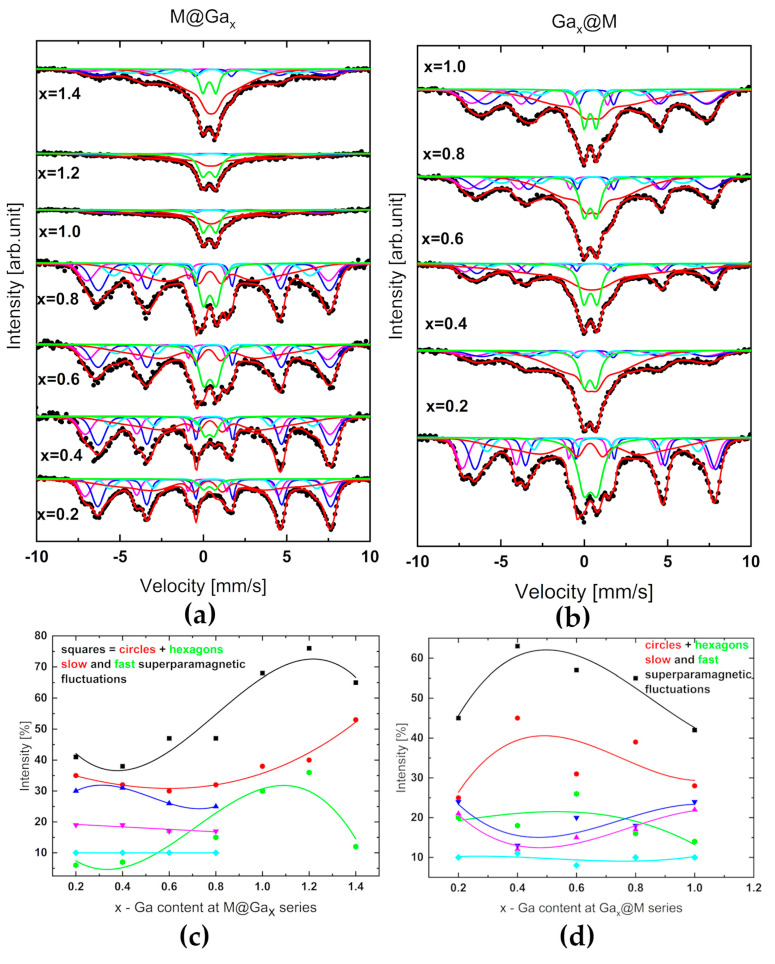
Mössbauer spectra (black dots) collected at room temperature for (**a**) M@Ga_x_ and (**b**) Ga_x_@M series of shell@core type nanoparticles. The percentage contributions of hyperfine magnetic fields associated with Td (pink triangle down) and Oh (blue triangle up) magnetic sublattices as well as slow (red circles) and fast (green hexagons) superparamagnetic fluctuations as the result of deconvolution of experimental spectra of (**c**) M@Ga_x_ and (**d**) Ga_x_@M series. For better readability, the fractions of fast and slow magnetic fluctuations were summed to the common percentage contribution (black squares). The lines are to guide the eye only.

The gallium low-doped magnetite systems predominantly showed a ferrimagnetic structure. Mössbauer spectroscopy allowed the analysis of the distribution of hyperfine magnetic fields associated with the Fe atoms in the sublattice of the spinel structure and superparamagnetic fluctuations. Nanosystems of Ga_x_@M series revealed superparamagnetic fluctuations (Figure 6a–d) much earlier with increasing x than M@Ga_x_-type nanoparticles.

Figure 6a,b illustrate experimental spectra as the result of the superposition of hyperfine magnetic field distribution and superparamagnetic origin. It is worth noting that the strongest contribution of superparamagnetism in the series on the right panel is observed for systems with Ga content in the range 0.4–0.8. The system with the content of gallium x = 0.6 was found to be optimal in terms of the heat capacity discussed later (Section 2.5).

According to Coey, who studied fine particles of γ-Fe_2_O_3_ with a diameter of 6 nm using ME [35] even in a magnetic field of 50 kOe, there is no complete ordering of the spins along the direction of the field. By analogy to other series of nanoferrites [43,44] the reason for this may be the existence of a disordered spin structure on the surface of the particles, occurring as a result of magnetic frustration caused by broken magnetic bonds, and the competition of antiferromagnetic exchange interactions of both magnetic sublattices (Th and Oh) of the inverse spinel structure. It is worth noting that the same effect was also observed in magnetic studies, Mössbauer spectroscopy, and inelastic neutron scattering carried out for CoFe_2_O_4_ and NiFe_2_O_4_ spinels [36,37,38]. In turn, the authors of the papers [46,47,48,49] suggested that the non-collinear spin structure is not a “pure” surface effect, but rather applies to the entire volume of the particle. A clear solution has not yet been found for this problem. The competition of the magnetic ordering of the core and the surface layer thus determines the state of the particle, which departs significantly from the simple assumption of a single-domain particle with a magnetic order analogous to that of the bulk material. The crystal symmetry disturbed at the particle boundary can propagate its effects from the surface layer deep into the core of the particle, and for a sufficiently small particle size, lead to its magnetic behavior determined almost entirely by the magnetic behavior of the surface.

It is intriguing that in both series, in the systems with the highest presented Ga concentration, i.e., M@Ga_1.4_ and Ga_1.0_@M, the magnetic order seems to be reconstructed.

### 2.5. SAR and Heat Cacapcity Measurements

The specific absorption rate (*SAR*) is a parameter that describes the rate at which energy is absorbed by a medium. This coefficient can also be defined as the capacity of a magnetic sample that has absorbed energy from a magnetic field (Equation (12)).

(12) $$SAR=\frac{c}{\varphi }\frac{\Delta T}{\Delta t}=\frac{P}{m},$$

where: c—specific heat of ferrofluid, φ—concentration of magnetic nanoparticles in ferrofluid, C—heat capacity, $\frac{\Delta T}{\Delta t}$—rate of temperature change over time, P—power deposited by magnetic nanoparticles, m—mass of nanoparticles in ferrofluid.

**Figure 7:**
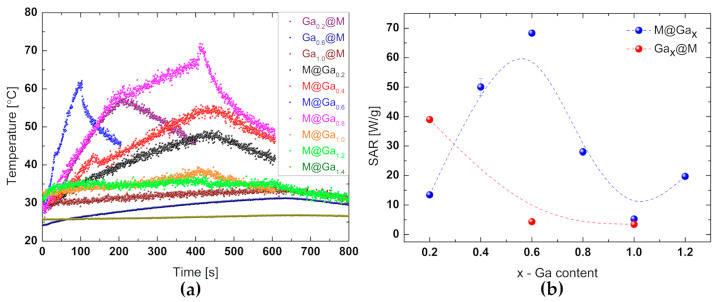
Heating characteristics of samples with a concentration of 10 mg/mL with gallium in the core and jacket (**a**) and SAR coefficients depending on the concentration of Ga (**b**).

Heating curves of particular sample can be seen in Figure 7a, while SAR estimation is shown in Figure 7b. For most of the tested systems, the heating time was about 400 s, then they were cooled down. Samples of Ga_0.2_@M and Ga_0.6_@M were heated to 100 or 200 s.

Figure 7a shows the heating characteristics of the nanosystems. The highest temperature was achieved by the system Ga_x_ = 0.6 in the core, which is correlated with the highest SAR (Table 3) result for the entire tested series.

On the base of calorimetric measurements, the specific absorption rates (SARs) were determined. It can be found that the higher SAR value was received for Table 3. Calorimetric studies confirmed the dependence of SARs on the concentration and location of gallium. An upward trend in these coefficients has been revealed, persisting in a gallium content of x = 0.6 in a core (Table 3 and Figure 7b). The obtained SAR values were at the level of those once characterizing nanoparticles with confirmed biomedical applications [50].

### 2.6. Effect of Nanoferrites on HeLa Cells and Fibroblasts In Vitro

**Figure 8:**
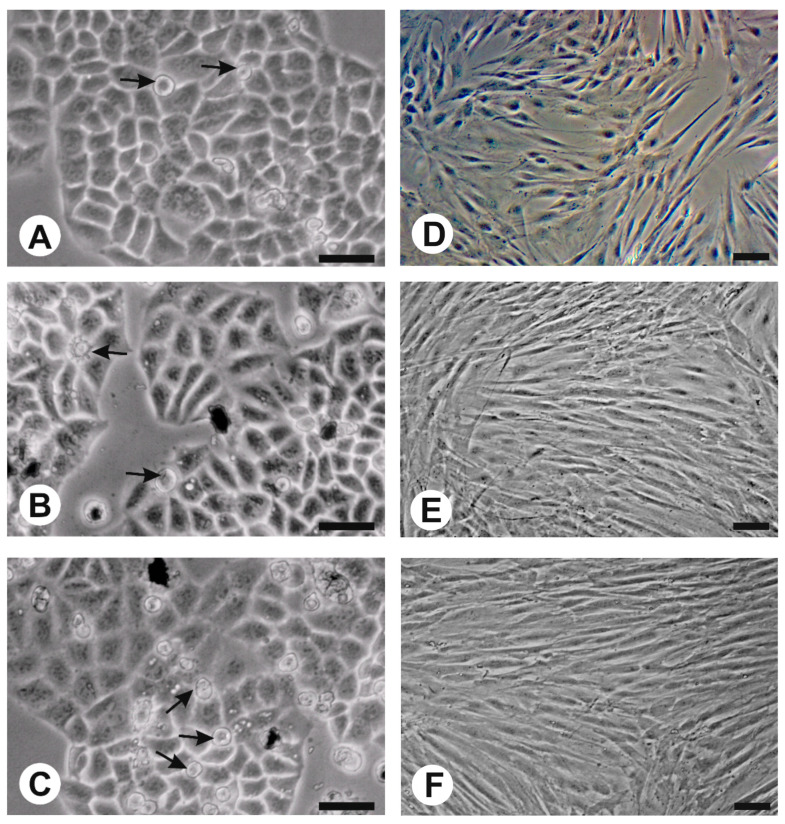
Morphology of cells undergoing experiment. (**A**)—HeLa control culture, (**B**)—HeLa culture with 0.01 mg/mL of M@Ga_0.6_, (**C**)—HeLa culture with 0.01 mg/mL of Ga_0.6_@M, all after 3 days of culture. (**D**)—Fibroblast control culture, (**E**)—Fibroblast culture with 0.01 mg/mL of M@Ga_0.6_, (**F**)—Fibroblast culture with 0.01 mg/mL of Ga_0.6_@M. The arrows indicate detaching cells, bar = 20 µm.

In vivo observation indicated that the experimental cultures with nanoparticles showed no major morphological differences in comparison with the control in the case of both HeLa cells and fibroblasts (Figure 8A–F). The only observed morphological change in HeLa cells was more cells which did not show adhesion to the bottom of the plate after three days of culture, especially with Ga_0.6_@M (Figure 8A–C).

**Figure 9:**
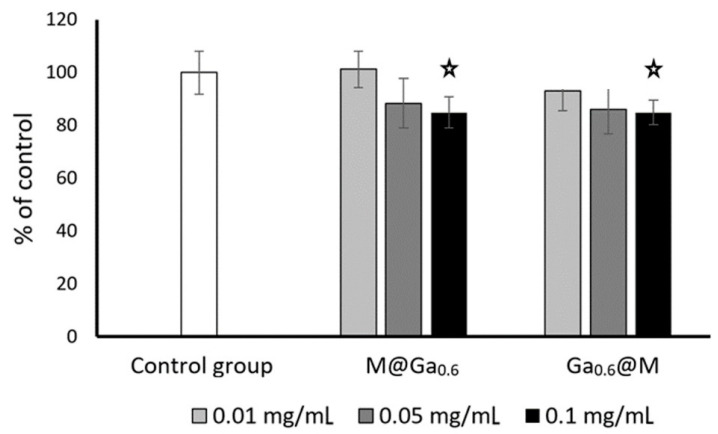
Changes in total amount of HeLa cells depending on the content and type of nanoparticles (% of control ± SE). The stars represent student’s *t*-test for a single sample = 100%, *p* < 0.01, n = 6.

**Figure 10:**
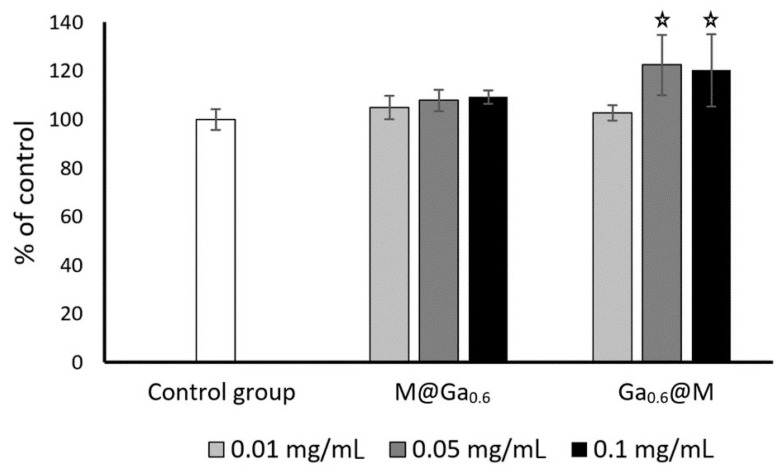
Changes in total amount of fibroblast cells depending on the content and type of nanoparticles (% of control ± SE). The stars represent student’s *t*-test for a single sample = 100%, *p* < 0.01, n = 6.

The initial quantity of cells in HeLa cultures was 1.5 × 10^5^ per well. After 3 days of incubation, the number of cells in control cultures reached ±4.1 × 10^6^ cells/mL. At the same time, experimental cultures containing 0.1 mg/mL of nanoparticles M@Ga_0.6_ and Ga_0.6_@M reached 3.96 × 10^6^ and 3.66 × 10^6^ cells/mL, respectively, which represented a decrease of about 10% in the number of cells compared to the control. A lower quantity of nanoparticles (0.01 and 0.05 mg/mL) did not cause significant changes in the rate of cell growth; however, it was noticed that there was a tendency for a concentration-dependent reduction in HeLa cells’ growth rate caused by both nanoparticles used (Figure 9). Contrary to the above-mentioned results, in the case of fibroblasts, stimulation of cell growth (approximately 20%) was observed in culture containing Ga_0.6_@M nanoparticles at the concentration of 0.05 and 0.1 mg/mL (Figure 10). M@Ga_0.6_ did not change the growth rate of fibroblasts. The initial concentration of fibroblast cells was 1.5 × 10^5^ per well. After 7 days of culture in control condition, 3.14 × 10^6^ cells/mL was found. At the same time, the number of fibroblasts increased to 3.85 × 10^6^ cells/mL and 3.68 × 10^6^ cells/mL under the influence of Ga_0.6_@M at the concentration of 0.05 mg/mL and 0.1 mg/mL, respectively.

HeLa cell viability was tested in every variant, reaching more than 95% in the case of the control culture and similar (95–93%) in cultures with nanoparticles after 3 days of incubation. In the case of the tested Ga_0.6_@M content, a slightly higher number of death cells was found at the concentration of 0.5 mg/mL compared to the control, while in the case of M@Ga_0.6_, the content of nanoparticles did not cause an increase in the number of dead cells (Table 4). In the case of control culture of fibroblasts, the viability of the cells was over 96% after 7 days. M@Ga_0.6_ did not change the cells’ viability in comparison to the control, regardless of the concentration, whereas Ga_0.6_@M, in contrast to the data obtained with HeLa cells, caused a slight decrease in cell viability at a concentration of 0.1 mg/mL in comparison to the control (Table 5).

Analyzing the result of the MTT test in the case of HeLa cells, a slight reduction was noticed in the metabolic rate in the culture with the highest content of Ga_0.6_@M (Table 6). In the case of M@Ga_0.6_, a slight decrease was observed of the metabolic rate at the concentration of 0.5 mg/mL (Table 6). The stronger effect of the metabolic rate increase, in comparison to that of HeLa cells, was found in the case of the fibroblast culture on the medium with the highest concentration of M@Ga_0.6_, which was accompanied by neither an increase in the rate of cell growth nor their survival. Ga_0.6_@M did not change fibroblasts’ metabolic rate (Table 7).

## 3. Materials and Methods

In this study, two series of shell@core magnetite nanoparticles, Ga_x_@M and M@Ga_x_, with 2.85–20 at. % gallium doping (x = 0.2–1.4) were used, where in one case the gallium ferrite coated the magnetite core, and in the other case the magnetite coated the gallium ferrite core. In order to prepare the magnetite core, 4 mmol of Fe(acac)_3_ was mixed with 1,2-hexadecanediol, oleylamine, and oleic acid in a high-boiling solvent (diphenyl ether). This mixture was heated to 259 °C (boiling point of diphenyl ether) in the presence of argon flow. In the next step, when the mixture was cooled down, a gallium ferrite shell was prepared on the resulting Fe_3_O_4_ core, e.g., with the stoichiometry of Ga_0.6_Fe_2.4_O_4_. For this purpose, 0.8 mmol of Ga(acac)_3_, 3.2 mmol of Fe(acac)_3_, oleylamine, and oleic acid were added to the obtained magnetite cores. The mixture was again heated to 259 °C in the presence of argon flow. When the solution cooled, the nanoparticle precipitation process was started by adding an excess of deoxygenated acetone. Acetone was exchanged 3 times, and then the precipitate was dried in the vacuum evaporator. In the case of coating gallium ferrite cores with magnetite, the procedure was analogous, with an appropriate change in the order of adding the substrates.

### 3.1. X-ray and Neutron Dyffraction

Room temperature (RT) XRD measurements were performed using Empyrean PANalytical powder diffractometer located at the Faculty of Physics University of Bialystok (FP, UB). A diffractometer was used working at 40 KV and 40 mA, using Mo_Kα_ radiation, λ= 0.7093187 Å in Bragg–Brentano geometry. A scattered intensity was recorded using a PixCel1D strip detector within a 2θ range from 5° to 55° and in a step-scanning mode with a step of Δ2θ = 0.026°. Pure LaB_6_ (660 C) powder standard sample was used to correct the data for instrumental broadening. Neutron diffraction spectra obtained using a time-of-flight instrument RTD (IBR-2, JINR, Dubna) [51] were normalized by calibrated scattering from a standard. To determine the shape of an incident neutron spectrum, the scattering from a vanadium sample was used. The diffraction patterns were analyzed using a Rietveld-type profile refinement method using the FullProf program [52] and HighScore software package 4.0 [53]. The full-profile Rietveld method leads to the average structure determination from the Bragg peak positions and intensities, and the microstructure determination (size–strain analysis) from the peak profile analysis [52,53]. The FullProf program allows the estimation of the average crystalline size and microstrain by averaging the values obtained for apparent size and maximum strain calculated for each crystal direction [hkl]. X-ray powder diffraction data peak profiles were modeled by the Thomson–Cox–Hasting modified pseudo-Voight function. Such peak profiles and the instrumental resolution function were used to obtain the apparent crystallite size.

### 3.2. TEM Images

Transmission electron microscopy (TEM) images were obtained at the Center of Synthesis and Analytical BioNanoTechno Methods at the Faculty of Chemistry University of Bialystok (FCh UB). The morphology and microstructure characterization of the particles were carried out using a FEI Tecnai G2 electron microscope operating at 200 kV.

### 3.3. SANS Investigations

SANS data were collected using a YuMO spectrometer operating on IBR-2. All samples were measured at four temperature points in the range between 20 °C and 50 °C. The particles’ shapes, sizes, dispersion coefficients, and morphologies were analyzed using the SAS-view program [54]. In order to most precisely determine SANS data descriptions, the necessity to use three different shape models was presented and justified, as described in detail in Section 2.3.

### 3.4. Mössbauer Spectroscpy

Mössbauer measurements were performed at room temperature on a standard spectrometer operating in the constant acceleration mode with a ^57^Co gamma source in an Rh matrix (FP, UB). The velocity scale was calibrated using α-Fe standard foil, also at room temperature. Mössbauer spectra were analyzed using the commercially available NORMOS package [55] using Voigt profile convolution of Lorenz and Gaussian shapes as a lines shape.

### 3.5. SAR and Heat Cacapcity Measurements

Calorimetric experiments under nonadiabatic conditions were performed using the magneTherm system (nanoTherics, Staffordshire, UK), while the analysis was performed by corrected slope method (CSM) [56,57]. According to the experimental apparatus, inside a 100.5 mL Styrofoam jacket, a 3 mL Eppendorf tube was filled with 2 mL of a ferrifluid sample. The frequency f = 532 kHz and intensity H = 15 kA/m were selected as the optimal parameters of the alternating magnetic field. The concentration of nanoparticles in the tested samples was 10 mg/mL. The solvent was diphenyl ether.

### 3.6. Effects of Nanferrites on Fibroblasts and HeLa Cells in In Vitro Culture

For evaluation of the impact of nanoparticles on cells, the in vitro culture method was used with HeLa (ATCC-CCL-2) cells as the cancer model and fibroblast cells (ATCC-CRL-2106) as the normal cells model. Before being added to the culture medium, nanoparticle powders were sterilized for 30 min in 70% ethanol and evaporated overnight to dryness at 60 °C. For preparation of nanoparticle suspension in culture medium, an ultrasonic processor Hielscher UP50H was used (0.5 s cycle, 100% amplitude by 60 s/3 min in ice). The content of both kinds of nanoparticles in separate culture medium was 0.1 mg/mL, 0.05 mg/mL, and 0.01 mg/mL. Control cultures of HeLa and fibroblast cells did not contain nanoparticles. All cultures were maintained in a NuAire NU-5820E incubator (37 °C, 5% CO_2_, 95% humidity) on 2 mL MEM (Sigma-Aldrich ref. no. M4655) medium, with 10% fetal bovine serum (Sigma-Aldrich ref. no. F7524) and antibiotics (penicillin 50 U/mL, streptomycin 0.05 mg/mL, Sigma-Aldrich ref. no. P0781). The initial inoculum was 1.5 × 10^5^ cells per well. Control cultures (without nanoparticles) and experimental variants (with nanoparticles) were grown in 12-well plates until the control variant reached 90% confluence (3 days in the case of HeLa cells and 7 days for fibroblasts). In the case of fibroblasts, the medium was changed after 3 days of culture. During the experiment, in vivo observations of cultures were carried out using an Olympus inverted contrast phase microscope (CKX 41).

For cell counting and viability estimation, an EVE^MT^ automatic cell counter (NanoEnTec Inc., Seoul, Republic of Korea) was used, with Trypan-blue staining [58] at a final concentration of 0.2% for 3 min.

The impact of nanoparticles on cell metabolism was evaluated using the MTT test [59] using tetrazolium salt (3-(4,5-dimethylthiazol-2-yl)-2,5-diphenyltetrazolium bromide, Sigma-Aldrich ref. no. M2128) at a final concentration in DPBS of 0.25 mg/mL (30 min incubation in 37 °C). The absorbance of the obtained solution was measured at λ = 570 nm using a Lambda E MWG AG BIOTECH plate reader. The results are presented as percent of control.

Data from six independent experiments were used for statistical evaluation. The results obtained from individual research methods were analyzed using the Shapiro–Wilk W-test to identify the normal distribution of data and the Levene L-test for verifying if the variances were homoscedastic. Since the obtained data showed a normal distribution and homoscedastic variances, the parametric Student’s t-test for a single sample = 1 (100%) was used to compare data expressed as percent of control. When data did not show a normal distribution, a nonparametric Mann–Whitney U-test was used to compare the average values of control and experimental groups.

## 4. Conclusions

Core-shell gallium nanoferrites were prepared from acetylacetonates. Using various techniques, it was possible to find a number of correlations between the morphological, structural, and magnetic parameters of the Ga_x_@M and M@Ga_x_ systems with x increasing to 1.4. Optimal biomaterials were selected.

All obtained nanostructures crystallize in the cubic inverse spinel type structure. Diffraction studies confirmed the homogeneity, ordering, and evident low-temperature magnetism of nanoparticles (neutron diffraction). TEM images of Ga_x_@M series disclosed a relatively weaker agglomeration effect, and in consequence, more effective dispersion in the diphenyl ether. Mostly, gallium-doped magnetite nanoparticles kept spherically symmetrical shapes in the whole range of the studied admixtures. A core–shell model was used successfully to develop the most SANS data. From this point of view, shape, size, and T_B_ values of gallium ferrites with a Ga content in the range of 0.6–0.8 are optimal. Complementary analysis of the diffraction intensities of (220) and (311) reflections indicate the preference for the location of gallium in the Td sublattice and presence of oxygen vacancies as the volume rather than the surface effect. Analysis of crystallite size as a function of gallium content in both series showed a consistent trend, with a minimum in the range above gallium content of 0.8.

In both series, systems with the highest concentration of Ga, i.e., M@Ga_1.4_ and Ga_1.0_@M, disclose reconstruction of magnetic order. The strongest contribution of superparamagnetism in the series of Ga_x_@M is observed for systems with a Ga content in the range of 0.4–0.8.

Calorimetric measurements of the studied nanosystems showed that these particles, subjected to an alternating magnetic field, have the ability to heat up quickly. In the case of M@Ga_x_ series, the system with x = 0.6 heated up the fastest and reached the highest SAR (68.32 ± 0.25) W/g. The increase in gallium content in the shell is accompanied by a downward trend in SAR.

Considering the biological properties of gallium-doped nanoparticles that were found, as well as their effects on both cancer cells (slight growth inhibition) and normal fibroblasts (small increase of growth rate), it can be concluded that the use of gallium as a dopant in the nanoparticle structure is a promising research direction. The results obtained indicate that, of the nanostructures obtained, Ga_0.6_@M show the highest potential for use in cancer thermotherapy. The physical and biological properties of these nanostructures make them an alternative to classical iron nanoparticles. Gallium-containing nanoparticles, while maintaining their magnetic properties, can have a lesser effect on the free radical damage to cells due to their limited iron content.

## Figures and Tables

**Table 1:** The oxygen position parameter according to Wyckoff notation 32 e: $\left(X_{O},X_{O},X_{O}\right)$ obtained during RT X-ray data refinements with appropriate Bragg agreement factors—*R_B_* ^1^.

Sample	X_0_	R_B_
M@Ga_0.2_	0.2488 ± 0.0009	1.63
M@Ga_0.4_	0.2495 ± 0.0002	1.55
M@Ga_0.6_	0.2504 ± 0.0003	2.59
M@Ga_0.8_	0.2485 ± 0.0007	2.29
M@Ga_1.0_	0.2483 ± 0.0005	3.62
M@Ga_1.2_	0.2461 ± 0.0005	5.51
M@Ga_1.4_	0.2506 ± 0.0009	2.62
Ga_0.2_@M	0.2459 ± 0.0001	2.94
Ga_0.4_@M	0.2493 ± 0.0004	1.87
Ga_0.6_@M	0.2491 ± 0.0005	2.34
Ga_0.8_@M	0.2520 ± 0.0001	3.71
Ga_1.0_@M	0.2533 ± 0.0006	3.79

^1^$R_{B}=100\frac{\sum_{k}\left|I_{k}^{obs}-I_{k}^{cal}\right|}{\sum_{k}I_{k}^{obs}}$.

**Table 2:** Set of nanoparticle’s radiuses obtained from SANS data collected at 40 °C.

Sample	Model	Radius of Gyration [Å]		T_B1_ [K]	T_B2_ [K]
		Core Radius	Shell Thickness		
M@Ga_0.2_	Guinier–Porod	25.2 ± 3.2			
M@Ga_0.4_	Core–shell sphere	91.4 ± 0.6	10 ± 2	137.59	247.67
M@Ga_0.6_	Core–shell sphere	88 ± 3	11 ± 3	128.05	235.15
M@Ga_0.8_	Core–shell sphere	44 ± 1	10 ± 1	20.78	
M@Ga_1.0_	Core–shell sphere	69 ± 5	9 ± 1	62.63	72.31
M@Ga_1.2_	Core–shell sphere	52 ± 2	11 ± 1	33.00	
M@Ga_1.4_	Core–shell sphere	51.6 ± 0.9	16.5 ± 0.9	41.68	
Ga_0.2_@M	Sphere	85.2 ± 0.2		81.62	
Ga_0.4_@M	Core–shell sphere	105 ± 7	12.1 ± 0.2	211.91	
Ga_0.6_@M	Core–shell sphere	54 ± 2	10.5 ± 0.6	35.41	
Ga_0.8_@M	Core–shell sphere	47 ± 2	18.1 ± 0.3	36.41	
Ga_1.0_@M	Core–shell sphere	97 ± 3	19 ± 1	205.99	

**Table 3:** SAR factors of gallium-doped nanosystems.

Sample	SAR [W/g]
M@Ga_0.2_	13.45 ± 0.19
M@Ga_0.4_	50.1 ± 2.9
M@Ga_0.6_	68.32 ± 0.25
M@Ga_0.8_	28.0 ± 1.3
M@Ga_1.0_	5.24 ± 0.17
M@Ga_1.2_	19.71 ± 0.26
M@Ga_1.4_	0.57 ± 0.01
Ga_0.2_@M	38.99 ± 0.08
Ga_0.6_@M	4.33 ± 0.19
Ga_1.0_@M	3.45 ± 0.33

**Table 4:** Comparison of quantity of dead cells in HeLa cells in control and nanoparticle-containing culture.

Content of Nanoparticles	Nanoparticles	
	M@Ga_0.6_	Ga_0.6_@M
	% of Dead Cells ± SE	
0.0 mg/mL (control)	4.51 ± 0.44	
0.01 mg/mL	4.7 ± 0.83	6.0 ± 0.91
0.05 mg/mL	5.0 ± 0.97	6.9 * ± 0.46
0.1 mg/mL	6.1 ± 0.72	6.5 ± 0.74

* Mann–Whitney U-test, *p* < 0.01. Values were compared with control group, n = 6.

**Table 5:** Comparison of quantity of dead cells in fibroblast cells in control and nanoparticle-containing culture.

Content of Nanoparticles	Nanoparticles	
	M@Ga_0.6_	Ga_0.6_@M
	% of Dead Cells ± SE	
0.0 mg/mL (control)	3.53± 0.31	
0.01 mg/mL	2.18 ± 0.39	2.80 ± 0.43
0.05 mg/mL	2.70 ± 0.48	2.40 ± 0.66
0.1 mg/mL	2.64 ± 0.55	1.76 * ± 0.54

* Mann–Whitney U-test, *p* < 0.01. Values were compared with control group, n = 6.

**Table 6:** Comparison of MTT test for HeLa cell cultures containing nanoparticles.

Content of Nanoparticles	Nanoparticles	
	M@Ga_0.6_	Ga_0.6_@M
	% of Control ± SE	
0.01 mg/mL	101 ± 3.48	98 ± 2.1
0.05 mg/mL	90 * ± 2.27	100 ± 1.35
0.1 mg/mL	103 ± 1.10	93 * ± 1.19

* Student’s *t*-test for a single sample = 1 (100%), *p* < 0.01, n = 6.

**Table 7:** Comparison of MTT test for fibroblast cell cultures containing nanoparticles.

Content of Nanoparticles	Nanoparticles	
	M@Ga_0.6_	Ga_0.6_@M
	% of Control ± SE	
0.01 mg/mL	98 ± 4.44	92 ± 3.79
0.05 mg/mL	123 ±11.73	93 ± 3.42
0.1 mg/mL	166 * ± 22.93	110 ± 5.81

* Student’s *t*-test for a single sample = 1 (100%), *p* < 0.01, n = 6.

## Data Availability

The data supporting this study’s findings are available on request from the corresponding author.
